# Estimating Physical Activity in Children Aged 8–11 Years Using Accelerometry: Contributions From Fundamental Movement Skills and Different Accelerometer Placements

**DOI:** 10.3389/fphys.2019.00242

**Published:** 2019-03-18

**Authors:** Michael J. Duncan, Clare M. P. Roscoe, Mark Faghy, Jason Tallis, Emma L. J. Eyre

**Affiliations:** ^1^School of Life Sciences, Coventry University, Coventry, United Kingdom; ^2^School of Human Sciences, University of Derby, Derby, United Kingdom

**Keywords:** motor competence, motor development, cut-points, indirect calorimetry, energy expenditure

## Abstract

Accelerometers are widely used to assess physical activity, but it is unclear how effective accelerometers are in capturing fundamental movement skills in children. This study examined the energy expenditure during different physical activities (PA) and calibrated triaxial accelerometry, worn at the wrist, waist and ankle, during children’s PA with attention to object control movement skills and cycling. Thirty children (14 girls) aged 8 to 11 years wore a GENEActiv accelerometer on their non-dominant wrist, dominant wrist, waist and ankle. Children undertook eight, 5-min bouts of activity comprising being lay supine, playing with Lego, slow walking, medium walking, medium paced running, overarm throwing and catching, instep passing a football and cycling at 35 W. VO_2_ was assessed concurrently using indirect calorimetry. Indirect calorimetry indicated that being lay supine and playing with Lego were classified as sedentary in nature (<1.5 METs), slow paced walking, medium placed walking and throwing and catching were classified as light (1.51–2.99 METs) and running, cycling and instep passing were classified as moderate intensity (>3 METs). ROC curve analysis indicated that discrimination of sedentary activity was excellent for all placements although the ankle performed better than other locations. This pattern was replicated for moderate physical activity (MPA) where the ankle performed better than other locations. Data were reanalyzed removing cycling from the data set. When this analysis was undertaken discrimination of sedentary activity remained excellent for all locations. For MPA discrimination of activity was considered good for waist and ankle placement and fair for placement on either wrist. The current study is the first to quantify energy expenditure in object control fundamental movement skills via indirect calorimetry in children aged 8–11 years whilst also calibrating GENEActiv accelerometers worn at four body locations. Results suggest throwing and catching is categorized as light intensity and instep kicking a football moderate intensity, resulting in energy expenditure equivalent to slow or medium paced walking or cycling and running, respectively. Ankle worn accelerometry appears to provide the most suitable wear location to quantify MPA including ambulatory activity, object control skills and cycling, in children aged 8–11 years.

## Introduction

Accelerometers are becoming the most widely used measure of physical activity (PA) in public health research (Vale et al., 2015) as they provide an objective assessment of energy expenditure and time spent in different intensities of PA (Crouter et al., 2018). Over the past decade there has also been increasing use of accelerometery to estimate PA in children (Rowlands et al., 2014; Crouter et al., 2018). Such methods provide objective data which is more reliable and valid in children than alternative methods such as self-report (Rowlands and Eston, 2007). Despite this, accelerometry is not without its challenges in relation to PA assessment. They are relatively expensive to use, compared to other methods of PA assessment, require more complex data handling and processing techniques to estimate PA and their accuracy in tasks requiring greater use of the upper body, cycling, or non-linear movement is not fully established (Rowlands and Eston, 2007). Given the increase in popularity of accelerometry as an assessment tool, there have also been considerable efforts made to calibrate accelerometer cut-points which is needed to more accurately estimate physical activity in pediatric populations (Ryan and Gormley, 2013; Phillips et al., 2014; Duncan et al., 2016; Roscoe et al., 2017).

Accelerometry derived cut-points are useful in determining the extent to which children meet current PA guidelines for health and, as a consequence, are widely used in prospective population based studies as a means to determine efficacy of PA interventions and to inform public health practice. Determination of cut-points that are specific to age group (e.g., children), model of accelerometer and wear location are critical in ensuring accuracy of PA assessment. Despite this, there remains a need to refine the accuracy of accelerometry to better understand the influence of PA on child health outcomes (Crouter et al., 2018). In particular, the choice of placement site can impact wear compliance and precision of the prediction equation for PA (Crouter et al., 2018). The process of, and activities used in, accelerometer calibration also has a meaningful influence on the precision of the accelerometer prediction equation where accelerometers more easily classify ambulatory based activity (Ryan and Gormley, 2013).

The waist has traditionally been the most commonly used placement site when using accelerometers to measure PA (Montoye et al., 2016). Waist worn accelerometers also tend to perform better than their wrist worn counterparts (Rowlands et al., 2014). Conversely, studies looking at participant compliance find much higher levels of compliance when accelerometers are wrist worn, especially in young children (Rowlands et al., 2014). More recently, the ankle has shown promise as an accelerometer placement site to obtain valid estimates of PA (Crouter et al., 2018). There are, however, only a limited number of studies that have used ankle placement, and to date, only one study has examined this issue in children. Previous work using adults have reported that ankle worn accelerometery performs poorly compared to waist worn accelerometry (De Vries et al., 2011), whilst others have shown the use of the ankle location was similar or better than waist or wrist worn locations for estimating energy expenditure (Kim et al., 2014; Hibbing et al., 2018). More recently Crouter et al. (2018) reported, using a sample of 8–15-year olds, that energy expenditure estimated from an ankle worn actigraph was not significantly different from that determined by indirect calorimetry and estimates of time spent in light, moderate and vigorous physical activity as well as sedentary behavior using both methods were comparable. Given the paucity of studies examining the utility of ankle-based accelerometer estimates of PA in children, and the fact that recent work by Crouter et al. (2018) did not compare ankle-based placement to other more commonly used locations, research examining this issue is needed to better clarify the optimal accelerometer placement for children’s PA assessment.

Another important influence on the process of accelerometer calibration relates to the activities which the accelerometer cut-points are derived from. The majority of studies have tended to use a procedure which includes periods of time supine, seated and in ambulatory activity executed on a treadmill (Ryan and Gormley, 2013; Phillips et al., 2014; Duncan et al., 2016; Roscoe et al., 2017). The premise for such procedures is that as locomotor activity is the predominant activity in an individual’s day, the validation of accelerometers during this activity is of primary importance (Welk, 2005). However, children’s PA tends to be sporadic and omnidirectional in nature (Rowlands and Eston, 2007) and thus, accelerometer cut points derived predominantly using locomotor activities may not accurately reflect the actual physical activity levels of children. In particular, fundamental movement skills such as throwing and catching are conceptualized as important and a regular feature of children’s PA (Holfelder and Schott, 2014). Recent research has suggested it is important to specifically understand how the repeated performance of various types of object control skills contributes to activity intensity as there are no published MET values associated with object control skills in children (Sacko et al., 2018). This type of intermittent movement is a noted limitation in accelerometry-based assessment of PA (Trost et al., 2005), and while accelerometer-based assessment of object control skills has been examined in older adults (Hooker et al., 2011), there appears to be no studies that have examined this issue in children. Likewise, cycling, another common movement skill in children is rarely examined in pediatric accelerometery calibration studies potentially leading to erroneous estimates of habitual physical activity when using cut-points that are not derived using a cycle-based activity within its protocol. Such observations have been noted in adult based studies (Mannini et al., 2013) where the stable position of the wrist during cycling may result in activity intensity being systematically misclassified during cycling activity when using wrist worn accelerometers. Such a criticism may also be leveled at waist worn monitors. Ankle worn accelerometery may be a more practical option that may better reflect activities such as cycling. However, no studies to date have examined this issue in children.

The current study sought to address key gaps in the literature by (a) examining the energy expenditure during object control skills in children, as related to ambulatory activity and; (b) calibrating triaxial accelerometry, worn at the wrist, waist and ankle, during children’s PA with particular attention to object control fundamental movement skills and cycling alongside the more traditionally used locomotor-based calibration protocol.

## Materials and Methods

### Participants

An opportunistic sample of 30 healthy, Caucasian, children (14 girls, 16 boys) aged between 8 and 11 years of age (9.4 ± 1.4 years) from central England took part in this study following institutional ethics approval, parental written informed consent and child assent. Mean ± SD of height, mass and body mass index (BMI), was 1.4 ± 0.4 m, 34.6 ± 8.6 kg and 17.6 ± 2.5 kg/m^2^, respectively. All children were involved in grassroots junior football as part of their recreational sports activities.

### Procedures

Participants wore a GENEActiv monitor (Brand name used with permission) on their non-dominant wrist, dominant wrist, and dominant waist, similar to other work (Routen et al., 2012) as well as an additional monitor placed on the dominant ankle. In the case of the dominant ankle, this was determined by asking the children were asked which leg they considered the leg they most used for kicking and then verifying this with their parents. Monitors were worn through the testing period. The GENEActiv has been described in detail previously (Esliger et al., 2011) but in brief, the GENEActiv is a lightweight triaxial accelerometer which provides raw acceleration data. In the work by Esliger et al. (2011) it was found to have high intra and interinstrument reliability (coefficient of variation = 1.8 and 2.4%, respectively), good criterion-referenced validity (*r* = 0.97) when compared to a multi-axis shaking table and high concurrent validity with the Actigraph GT1M accelerometer. Esliger et al. (2011) also reported that, irrespective of whether the accelerometer was worn at the wrist or hip, the GENEActiv could be used to distinguish between sedentary, light, moderate, and vigorous activity behavior in adults.

The GENEActiv was chosen as it provides three-axis raw accelerometry data from monitors that can be worn on multiple body locations. The GENEActiv is also capable of capturing high frequency data (up to 100 Hz) for multiple days (up to 7 days at 100 Hz or 45 days at 10 Hz) and is thus attractive for researchers interested in assessing free-living PA. In the current study the GENEActiv was set to record at 80 Hz and 1 s epochs. Throughout the testing procedure VO_2_ and VCO_2_ were assessed using a MetaMax 3B (Cortex Biophysik GmbH, Leipzig, Germany) breath by breath gas analyzer. Participants wore a junior face mask (Hans Rudolph) and the MetaMax was calibrated with gasses of known concentration each day prior to commencing testing. All testing took place in the morning (9am–12pm). Prior to beginning the protocol, each participant was fully familiarized with the treadmill being used in the study (Woodway Inc., Wisconsin, United States).

After briefing and being fitted with the GENEActiv monitors and gas analyzer, each participant performed a series of activities reflective of different levels of PA. These were lying supine, seated and playing with Lego, slow walking, medium walking, and a medium paced run. These were performed in order as per prior work by Phillips et al. (2014). Participants then performed bouts of overarm throwing and catching a standard size tennis ball, instep passing a football (Size 3) and cycling (Lode Corival Pediatric, Lode BV, Groningen, Netherlands). All activities were performed for 5 min with a 5-min rest in between. Using previous protocols (Puyau et al., 2002; Ryan and Gormley, 2013) as guidelines, walking and running speeds were set at 3, 4.5, and 6.5 kmph^-1^ to represent slow, medium pace walking and running, respectively. Cadence for overarm throwing and catching and passing a football was set to ensure one complete action (e.g., a throw or football pass) was completed every 3 s. Specific instructions were given to the children in respect to each motor skill followed by a demonstration of each activity. For throwing participants were instructed to, rotate their hips and shoulders to the point where their non-throwing arm faced 90 degrees from their starting position, to transfer weight by stepping forward with their dominant foot prior to ball release and then to follow through beyond ball release diagonally toward the non-preferred side. When catching, the children were asked to move their arms in preparation with hands in front of the body and elbows flexed. To step forward with arms extended, reaching for the ball as it arrived and to only use the hands to catch. For instep passing the children were instructed to take a step forward immediately prior to ball contact with the non-kicking foot placed alongside or slightly behind the ball and to pass with the instep of the foot only.

### Data Processing

Upon completion of the protocol, each participant’s accelerometer and calorimetry data was downloaded and stored on a computer. The first and last minute of each bout were discarded leaving a 3-min period for analysis. This ensured that MET values for each bout were at the required intensity and is consistent with prior work (Phillips et al., 2014; Roscoe et al., 2017). Using the GENEActiv post processing software (Version 2.9), the raw 80 Hz signal from all three axes were summarized into a single vector magnitude (gravity subtracted) (SVM gs), congruent with prior work by other authors (Esliger et al., 2011; Phillips et al., 2014; Roscoe et al., 2017). The correction for gravity was undertaken to focus the outcome variable on dynamic rather than static accelerations, as recommended by Esliger et al. (2011), and used by prior authors (Phillips et al., 2014; Roscoe et al., 2017).

Data were saved in raw format as binary files and then data for each wear location were summed into a signal magnitude vector (gravity subtracted) expressed in 1 s epochs, as is conventional (Esliger et al., 2011; Phillips et al., 2014).

The VO_2_ values were then converted into METs using age-specific values (Harrell et al., 2005) and coded into one of four intensity categories (sedentary <1.5 METs), light (1.5–2.99 METs), moderate (3–5.99 METs) and Vigorous (>6 METs). However, on inspection none of the activities undertaken by the participants resulted in MET values in excess of 6. Data were then subsequently recoded into three intensity categories reflecting sedentary, light and moderate PA (MPA).

### Statistical Analysis

Prior to analysis data were checked for normality which confirmed that data were non-normal via the Shapiro-Wilk test (all *P* < 0.05). As a consequence Spearman’s rank correlations were employed to examine criterion validity of the GENEActiv output at each wear location and METs. Following this, separate Spearman’s correlations were performed between METs at each intensity (sedentary, light, moderate) and accelerometer counts at each wear location in order to provide greater clarity of the validity of the GENEActiv output at each intensity of activity. Receiver operating characteristic (ROC) curve analysis was undertaken (Jago et al., 2007) to determine SB and MPA cut-points. The area under the curve (AUC) was calculated for each analysis as a measure of diagnostic accuracy with AUC values of; ≥0.90 considered excellent, 0.80–0.89 good, 0.70–0.79 fair, and <0.70 poor (Metz, 1978). ROC curve analysis was conducted as described previously (Esliger et al., 2011; Phillips et al., 2014) and cut-points that maximized sensitivity (Se) and specificity (Sp) were derived (Perkins and Schisterman, 2006). In line with prior work, AUC was determined for SB and MPA leaving accelerometer counts that fell between the sedentary and MPA cut-points were then classified as light PA, in line with prior work (Phillips et al., 2014). Cut-points for light PA were classed as those higher than SB but lower than MPA but did not require AUC, Se or Sp values to be determined as per Phillips et al. (2014). These are subsequently labeled as not applicable (NA) in Tables 1, 2. ROC analysis was undertaken using the Statistical Package for Social Sciences (SPSS, version 24). Cut-points reflected recommendations that the lower Se or Sp values should be ≥60% (Lugade et al., 2014). This prioritization approach minimizes the risk of individuals being misclassified in the target behavior and is common in accelerometer calibration (Mackintosh et al., 2012) and fitness standards research (Welk et al., 2011).

**Table 1:** Spearman’s rank correlations between GENEActiv output and METs during sedentary, light and moderate intensity activities with and without cycling removed from analysis (^∗^*P* < 0.01).

	Non-dominant wrist	Dominant wrist	Waist	Ankle
**Cycling included**
Sedentary	0.177^∗^	0.154^∗^	0.228^∗^	0.429^∗^
Light	0.110^∗^	0.114^∗^	0.120^∗^	0.105^∗^
Moderate	0.530^∗^	0.542^∗^	0.508^∗^	0.611^∗^
**Cycling excluded**
Sedentary	0.268^∗^	0.371^∗^	0.099^∗^	0.489^∗^
Light	0.091^∗^	0.061^∗^	0.129^∗^	0.182^∗^
Moderate	0.413^∗^	0.443^∗^	0.480^∗^	0.689^∗^


**Table 2:** Sensitivity, specificity and area under the curve and resultant cut-points for each GENEA monitor.

Intensity	Location	AUC	95% CI	Sensitivity	Specificity	Cut-point (gs)
**Sedentary**
	Non-dominant wrist	0.901	0.891–0.911	87.6	83.4	4.8
	Dominant wrist	0.912	0.903–0.922	89.1	77.7	5.3
	Waist	0.934	0.926–0.942	88	94.6	4.3
	Ankle	0.977	0.974–0.981	95.3	85.7	4.4
**Light**
	Non-dominant wrist	NA	NA	NA	NA	4.9–11.99
	Dominant wrist	NA	NA	NA	NA	5.4–14.6
	Waist	NA	NA	NA	NA	4.4–8.2
	Ankle	NA	NA	NA	NA	4.5–129.1
**MPA**
	Non-dominant wrist	0.669	0.650–688	79.5	60.6	12.0
	Dominant wrist	0.661	0.642–0.680	79.3	60.9	14.7
	Waist	0.742	0.724–0.759	81.6	64.7	8.3
	Ankle	0.869	0.858–0.880	98.8	73.8	129.2


## Results

**FIGURE 1:**
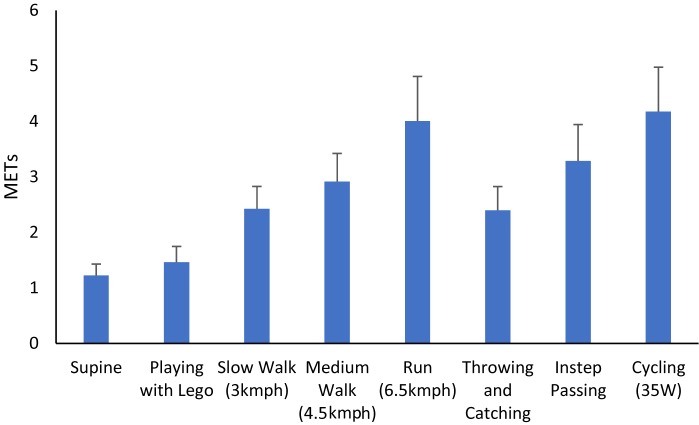
Mean ± *SD* of METs in activities undertaken reflective of different levels of PA.

Results from indirect calorimetry are presented in Figure 1. When the child was lay supine and playing with Lego were classified as sedentary in nature (<1.5 METs), slow paced walking, medium placed walking and throwing and catching were classified as light (1.51–2.99 METs) and running, cycling and instep passing were classified as moderate intensity (>3 METs).

Spearman’s rank correlations indicated significant weak relationships between METs and GENEActiv counts at the non-dominant wrist (*r* = 0.415, *P* = 0.0001), dominant wrist (*r* = 0.458, *P* = 0.0001), waist (*r* = 0.505, *P* = 0.0001), and a moderate relationship at the ankle (*r* = 0.752, *P* = 0.0001). When analysis was rerun removing cycling-based activity the strength of the relationship between METs and GENEActiv counts at each location increased. Spearman’s correlation values between METs and GENEActiv counts were *r* = 0.715 (*P* = 0.0001) for the non-dominant wrist, *r* = 0.720 (*P* = 0.0001) for the dominant wrist, *r* = 0.774 (*P* = 0.0001) for the waist and *r* = 0.790 (*P* = 0.0001) for the ankle, demonstrating appropriate criterion validity between GENEActiv output and overall activity. Subsequent analysis examining the association between GENEActiv counts and METs at each intensity of activity revealed a similar pattern to that of the overall activity where significant weak to moderate relationships were found for all wear locations within sedentary activity, light activity and moderate activity (all *P* < 0.01, See Table 1) irrespective of whether cycling was included or excluded in the analysis. The strongest associations between METs in each intensity and GENEactiv output was observed for the ankle placement with the exception of light METs when cycling was included in the analysis where the waist performed marginally better than the other wear locations.

Receiver operating characteristic curve analysis for the GENEActiv monitors worn at the non-dominant wrist, dominant wrist, waist and dominant ankle were able to successfully discriminate different intensities of activity. Sensitivity, specificity, AUC and resultant cut-points for each GENEA monitor are presented in Table 2. Discrimination of sedentary activity was excellent although the ankle performed better than other locations. This pattern was replicated for MPA where the ankle location performed better than other locations. Ankle discrimination was considered good, discrimination at the waist fair but discrimination at non-dominant and dominant wrist was considered poor. Ankle worn GENEActivs had the highest sensitivity for sedentary behavior and MPA. Waist worn GENEActivs had the highest specificity for sedentary behavior whereas the highest specificity for MPA was for ankle worn GENEActivs.

Considering the recognized issues where the stable position of the wrist during cycling resulting in activity being misclassified when using wrist worn accelerometers, data were reanalyzed with cycling activity removed from the analysis (See Table 3). When this subsequent analysis was undertaken, discrimination of sedentary activity remained excellent for all locations, although waist placement performed slightly better than the ankle or either wrist. For MPA activity, discrimination of activity was considered good for waist and ankle placement and fair for placement on the non-dominant and dominant wrist. There was similar sensitivity for all monitor locations for sedentary activity, but the wrist worn GENEActiv had lower specificity for sedentary activity compared to wrist and ankle locations. For MPA wrist worn monitors had lower sensitivity and specificity than waist and ankle worn monitors.

**Table 3:** Sensitivity, specificity and area under the curve and resultant cut-points for each GENEA monitor with cycling removed from analysis.

Intensity	Location	AUC	95% CI	Sensitivity	Specificity	Cut-point
**Sedentary**
	Non-dominant wrist	0.974	0.969–0.979	98.3	82.7	8.9
	Dominant wrist	0.977	0.973–0.981	97.8	80.2	11.5
	Waist	0.993	0.969–0.978	99.2	88.1	6.4
	Ankle	0.974	0.969–978	95.9	88	4.4
**Light**
	Non-dominant wrist	NA	NA	NA	NA	9.0–34.6
	Dominant wrist	NA	NA	NA	NA	11.6–29.4
	Waist	NA	NA	NA	NA	6.5–30.5
	Ankle	NA	NA	NA	NA	4.5–121.3
**MPA**
	Non-dominant wrist	0.798	0.783–0.813	87.7	73.1	34.7
	Dominant wrist	0.776	0.759–0.792	85.7	71.4	29.5
	Waist	0.861	0.849–0.873	92.1	71.0	30.6
	Ankle	0.856	0.844–0.869	96	74.0	121.4


## Discussion

This study provides novel data quantifying the energy expenditure in fundamental movement skills and calibrating the GENEActiv accelerometer in children aged 8–11 years across four different wear locations and with particular attention to object control fundamental movement skills and cycling. The quantification of energy expenditure during fundamental movement skills in children has previously not been reported and there are no directly established (e.g., via indirect calorimetry) MET values associated with object control skill performance in children. The current study is the first to provide this insight and addresses recent calls for this information to be provided (Sacko et al., 2018).

The results of the present study suggest that participants’ metabolic expenditure while performing object control skills was light (throwing and catching) to moderate (instep kicking) in nature. These data suggest that practicing object control skills in the form of instep football kicking would be classified as MPA and illustrates that repetitive performance of fundamental movement skills can contribute to achieving recommended guidelines for physical activity in children. Given the paucity of studies on this topic in children it is difficult to draw conclusions with prior work. However, recent work by Sacko et al. (2018), conducted in adults, reported execution of blocked trials of kicking, throwing and striking executed with maximal effort and at different cadences, produced metabolic expenditure that was moderate to vigorous in nature. In the current study, and using a different protocol, only instep kicking entered the moderate threshold with throwing and catching being of light intensity.

The GENEActiv accelerometers at each wear location demonstrated acceptable criterion validity with METs based on both the results from Spearman’s correlations, showing the relationships between GENEActiv output and MET values, and AUC data from ROC analysis which gives an indication of classification accuracy of the GENEActiv output to the criterion (METs). However, the strength of association of Spearman’s correlation was lower when cycling activity was included in the protocol. This was particularly the case for accelerometers worn at the wrist and the waist. The inclusion of cycling with accelerometer calibration protocols has been a point of debate. Cycling is a lifetime physical activity which is health enhancing but results in minimal movement at the waist and wrist, compared to other more ambulatory activities (Mannini et al., 2013). This often results in misclassification of cycling activity by accelerometers worn at the wrist and waist (Welch et al., 2013). In the present study the strength of association between METs and accelerometer counts from the wrist and waist were weaker when cycling was included compared to when it was removed from the protocol. Irrespective of protocol, the strongest association and therefore best criterion validity, between accelerometer counts and METs was for the ankle wear location. This observation was consistent when total METs was examined and when separate analysis was conducted for SB, LPA, and MPA. Such a finding aligns with recent work by Crouter et al. (2018) which also highlighted the utility of ankle worn accelerometry for estimating physical activity in youth.

Receiver operating characteristic curve analysis also supports the validity of ankle worn accelerometry given that the largest AUC values were found for MPA assessment at this wear location. Where the highest AUC were observed for the ankle in SB and MPA (See Table 2.). The results of the present study extend prior work in this area (e.g., Phillips et al., 2014; Duncan et al., 2016; Roscoe et al., 2017) that have used calibration activities involving predominantly ambulatory activity and examined wrist and waist worn devices. These aforementioned studies provide distinct AUC data and subsequent cut-points for the waist and wrist based on activities that are, arguably, the easiest for an accelerometer to quantify. Children’s movement patterns are omnidirectional and rarely comprise solely of walking/running type physical activity. In the current study we included cycling, given its role as a lifelong health enhancing physical activity, and two object control skills, throwing and catching and instep kicking. These object control skills were included given their importance in participation in physical activity (Morgan et al., 2013). For this reason, accelerometer cut-points for use in pediatric samples should be sensitive to detecting these forms of movement. Without considering these types of activities there is likely to be a drastic underestimation of energy expenditure in activities that include object control skills such as football, basketball, and racquet sports (Rowlands and Stiles, 2012).

No study to date has examined the utility of GENEActiv accelerometers worn at the ankle to classify PA in children. It is therefore difficult for the present study to draw comparisons with prior studies. However, the current study is supportive of work conducted by Crouter et al. (2018) suggesting ankle worn accelerometry (Actigraph) has potential to measure physical activity accurately in youth. Unfortunately, Crouter et al. (2018) did not compare ankle worn accelerometery to estimations derived from other locations. Other work with youth and using Actigraph accelerometers (De Vries et al., 2011) has suggested the waist location may be better than the ankle in predicting adult physical activity using artificial neural networks, whereas research using the Actical accelerometer (Heil, 2006) has reported no differences in energy expenditure estimation from devices worn at the wrist, ankle or waist. In the present study, however, ankle worn accelerometry appears to offer a more accurate means to estimate physical activity using the GENEActiv accelerometer when cycling and object control skills are also considered. The cut-points presented for the ankle, wrist and waist placements are not, however, interchangeable as body segments will move with different amounts of acceleration for different intensity movements. One-second epoch were also used, as is conventional in calibration studies (e.g., Phillips et al., 2014; Roscoe et al., 2017) and allows for upscaling to larger epochs which may be more useful in monitoring of habitual activity over multiples days.

The data presented here are based on activities conducted in a laboratory setting. This is a needed first step to establish energy expenditure in the movement skills of interest and to calibrate the accelerometer against breath-by-breath indirect calorimetry derived energy expenditure. A useful next step for researchers is to apply the cut-points derived in the current study in free living physical activity in children providing cross-validation of the cut-points presented in this study. In practical terms using the cut-points we present that include cycling within the calibration may be better reflective of the diversity of activities that children undertake habitually.

It would be beneficial to also understand if the cut-points presented here correctly classify different object control skills as light (throwing and catching) or moderate (kicking) in nature. Cross-validation of the current cut-points is needed to answer this question. Subsequent use of machine learning approaches to activity classification may also be an interesting technique to answer this question. In comparison to prior work, only Phillips et al. (2014) present cut-points for the age of population we examined in the present study, and only examined the wrist and waist locations. The cut-points we present for SB for those locations are similar to those reported by Phillips et al. (2014). However, the cut-points for MPA are slightly lower than those reported by Phillips et al. (2014). This discrepancy is not unexpected as the work by Phillips et al. (2014) relied primarily on treadmill based activity alongside a primarily upper body and linear activity on the Nintendo Wii, whereas the protocol employed in the current study comprised more varied activities, typical of children’s habitual PA. Comparing accelerometer counts worn at all four locations against estimates of energy expenditure from direct observation or, if possible, expired gas, in settings where fundamental movement skills are typically performed (e.g., children’s organized sports), would be a useful future research study. In the current study, participants were children who engaged with grassroots football. In this way we sought to pragmatically recruit children who were engaged in activity that necessitated use of fundamental movement skills as part of regular recreation. However, the results presented here are therefore indicative of children who had “good” motor competence and were all within “healthy” BMI based weight status categories. Level of technical skill may contribute to total energy expenditure (Sacko et al., 2018) and it is possible that children who are not fully competent in their fundamental motor skills will expend more energy for the same movements and young sports performers who are highly technically proficient may be more economical in their movement patterns resulting in less energy expended for the same movement. To date this issue has not been investigated in the context of assessing physical activity using accelerometry. We are also conscious that this study evidences utility of accelerometers worn at different locations. Although ankle worn accelerometry produced better classification of physical activity we did not examine any issues around compliance to ankle worn wear protocols. Compliance to wear protocols in habitual physical activity studies with children are also important. Prior work (Rowlands et al., 2014) has suggested higher wear compliance for wrist worn, compared to waist worn accelerometry with children. Other research (Tudor-Locke et al., 2015) has suggested acceptable compliance rates using ankle worn accelerometry over 24 h. Future research examining this issue using the GENEActiv accelerometer would be useful in translating the results of the current study into wider use for multi day assessment of physical activity. In practical terms, understanding how children respond to ankle worn accelerometry when worn over multiple days would be useful in establishing whether this is a viable alternative to the more commonly used wrist and waist worn protocols for the assessment of habitual PA. It is important to note that the protocol employed in the present study did not result in children undertaking energy expenditure of a vigorous intensity. Therefore, the cut-points established represent the threshold for MPA only. While the MPA threshold is essential for classifying whether children meet current physical activity guidelines, understanding differentiation of moderate and vigorous physical activity would be a useful next step. Related to this point, the activities employed in the current study to represent sedentary behavior comprised being lay supine and seated playing with lego. Inclusion of standing as a discrete sedentary behavior would also have been useful given that standing is sedentary behavior recognized as distinct from sitting or lying (Barone Gibbs et al., 2015). The time commitment and physical demand needed by children to undertake the current protocol did not, however, permit us to include additional activities or treadmill speeds. The safety aspect of asking children to run at faster speeds than those used in the present study also needs to be considered by future researchers, as in the current study requesting participants to run at any faster speed than was used was not feasible.

This study extends the literature in the area of physical activity assessment by quantifying energy expenditure in object control fundamental movement skills via indirect calorimetry in children aged 8–11 years and also calibrating the GENEActiv accelerometer during PA including object control skills and cycling and when worn at different body locations. The results of the current study suggest throwing and catching is categorized as of light intensity and instep kicking a football moderate intensity, resulting in energy expenditure equivalent to slow or medium paced walking or cycling and running, respectively. GENEActiv accelerometers demonstrated acceptable criterion validity although, when cycling was considered, validity of wrist and waist worn accelerometers was lower. Ankle worn accelerometry appears to provide the most suitable wear location to quantify MPA including ambulatory activity, object control skills and cycling, in children aged 8–11 years.

## Ethics Statement

The research presented in this manuscript was approved by the institutional ethics committees of Coventry University and the University of Derby and adhered in full to the Declaration of Helsinki in the treatment and use of human participants in research studies.

## Author Contributions

MD conceived the study, collected data, performed data processing and analysis, and drafted and edited the manuscript. CR and MF collected data, performed data processing, and edited the manuscript. JT conceived the study, drafted and edited the manuscript. EE conceived the study, collected data, and drafted and edited the manuscript. MD, CR, MF, JT, and EE approved the final version.

## Conflict of Interest Statement

The authors declare that the research was conducted in the absence of any commercial or financial relationships that could be construed as a potential conflict of interest.
